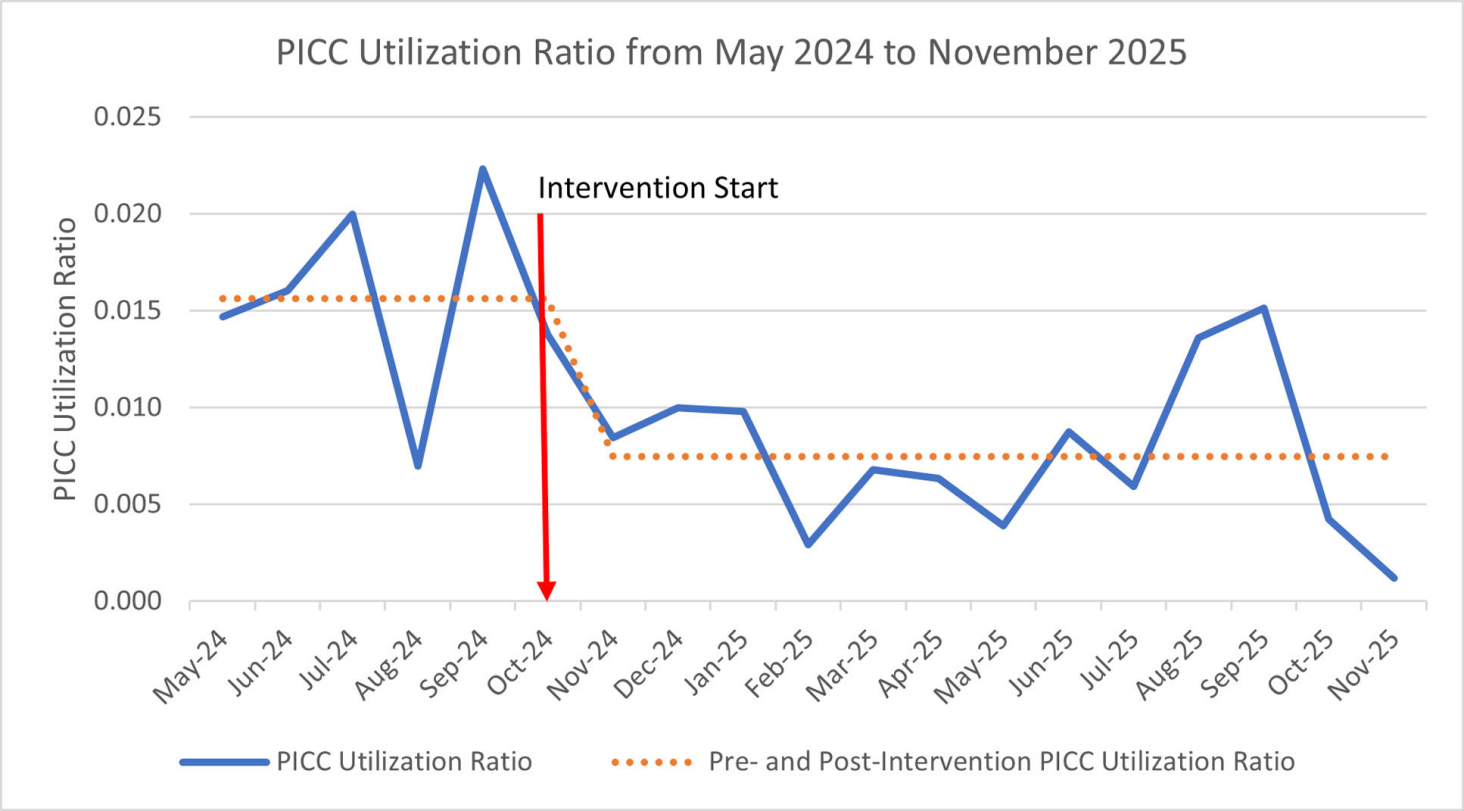# 357 Candida auris Screening Strategies Across a Multi?Hospital System

**DOI:** 10.1017/ash.2026.10695

**Published:** 2026-06-23

**Authors:** Jessica Gamboa, Bency Lukose, Musaddiq Waheed

**Affiliations:** 1 Infection Prevention and Management Associates; 2 HCA Healthcare; 3 HCA Houston Healthcare Southeast

## Abstract

**Background** Catheter associated bloodstream infections (CABSIs) remain a preventable healthcare-associated infection, with prolonged central venous catheterization posing a significant risk. Peripherally inserted central catheters (PICCs) are increasingly used in hospitalized patients due to convenience and a lower infection risk compared with nontunneled central venous catheters. Despite their benefits, PICCs are associated with complications, including thrombosis, phlebitis, infiltration and CABSI. Complications can delay treatment, prolong hospitalization and increase financial burden on healthcare facilities. Ensuring appropriate PICC use and considering less invasive alternatives can reduce patient harm and healthcare costs. Methods From May to October 2024, infection prevention team identified sustained high utilization of PICCs in the adult inpatient units. The infection prevention team organized and conducted a multidisciplinary meeting with the PICC team, Dietary, Pharmacy, and Nursing partners to identify opportunities to reduce unnecessary PICC use. Lack of a formal review for PICC insertion appropriateness and limited assessment of PICCs present on arrival were identified as main opportunities. In October 2024, the team implemented standardized review for all PICC indications and PICC line insertion request. The intervention included a multidisciplinary evaluation of all PICC lines. The review included daily assessment of PICC necessity, potential for device de-escalation and removal, and review of medication and parenteral nutrition orders for potential transition to formulations compatible with midlines or other lower risk access devices. Daily huddles facilitated communication regarding line necessity and escalation of barriers. Results Hospital-wide central venous catheter (CVC) utilization decreased 15.5% from a standard utilization ratio (SUR) of 0.653 in the baseline period, to 0.552 in the 6-month post-implementation period (p-value < 0 .001). Decreased CVC utilization was sustained with a SUR of 0.527 through November 2025. Furthermore, PICC Utilization decreased 52.5% from a utilization ratio of 0.016 to 0.0074. Conclusion PICC lines were frequently overutilized, contributing to unnecessary exposure to central venous access risks. Key opportunities included expanding midline use for short-duration therapies and peripheral administration of appropriate parenteral nutrition formulations. Implementation of a multidisciplinary PICC review process significantly reduced PICC utilization and decreased potential CABSI risk.

**Figure:**